# A web-based dashboard for *RELION* metadata visualization

**DOI:** 10.1107/S2059798323010902

**Published:** 2024-01-24

**Authors:** Nayim González-Rodríguez, Emma Areán-Ulloa, Rafael Fernández-Leiro

**Affiliations:** a Spanish National Cancer Research Centre (CNIO), Melchor Fernández Almagro 3, 28029 Madrid, Spain; b Department of Cell and Chemical Biology, Leiden University Medical Center, Leiden, The Netherlands; Rutherford Appleton Laboratory, United Kingdom

**Keywords:** cryo-electron microscopy, *RELION*, ice thickness estimation, graphical user interface, web-based cryo-EM tools

## Abstract

Cryo-electron microscopy (cryo-EM) has witnessed radical progress in the past decade, driven by developments in hardware and software. While current software packages include processing pipelines that simplify the image-processing workflow, they do not prioritize the in-depth analysis of crucial metadata, limiting troubleshooting for challenging data sets. The widely used *RELION* software package lacks a graphical native representation of the underlying metadata. Here, two web-based tools are introduced: *relion_live.py*, which offers real-time feedback on data collection, aiding swift decision-making during data acquisition, and *relion_analyse.py*, a graphical interface to represent *RELION* projects by plotting essential metadata including interactive data filtration and analysis. A useful script for estimating ice thickness and data quality during movie pre-processing is also presented. These tools empower researchers to analyse data efficiently and allow informed decisions during data collection and processing.

## Abstract 2

*relion_live.py* and *relion_analyse.py*, two web-based tools to enhance cryo-EM data processing in *RELION*, are introduced, providing an interface for real-time feedback on data collection and simplified interpretation of metadata. Additionally, an analytical script for ice-quality estimation is provided, empowering researchers to make informed decisions to improve data quality and accessibility in cryo-EM.

## 1. Introduction

Cryo-electron microscopy (cryo-EM) has undergone rapid development in the last decade, mostly due to the combination of advances in microscope stability, the development of direct electron detectors and improvements in data collection and processing software. As a result of these developments, which are referred to as the ‘resolution revolution’ (Kühlbrandt, 2014 ▸), there has been an exponential increase in the number of cryo-EM structures deposited in the Protein Data Bank (PDB; Henderson & Hasnain, 2023 ▸). Early image processing software packages developed for structure determination by single-particle analysis (SPA) relied heavily on the ability of the user to interact with the software via scripting, the command line and manual modification of text files. Currently, the most popular software packages include graphical user interfaces (GUIs; de la Rosa-Trevín *et al.*, 2016 ▸; Scheres, 2012 ▸; Fernandez-Leiro & Scheres, 2017 ▸; Tegunov & Cramer, 2019 ▸; Punjani *et al.*, 2017 ▸) that make cryo-EM accessible to non-experts. This has contributed to the increasing popularity of cryo-EM as a structural characterization technique for macromolecules.

The emergence of GUIs led to the simplification and automatization of processing pipelines, which restricts access to processing metadata and makes its manipulation cumbersome. However, inspecting and interacting with the metadata can be particularly useful in troubleshooting challenging cases. The metadata generated during image processing are stored in text files. These link individual files (either movies, micrographs, particles or volumes) with numerical values that contain all of the information produced during processing, *i.e.* contrast transfer function (CTF) information, particle coordinates and shifts, angular assignments, class assignments and many other parameters. *RELION* (Scheres, 2012 ▸), one of the most popular software packages for cryo-EM structure determination, uses the STAR file convention (Hall, 1991 ▸) to collect all processing metadata, lacking options for graphical representation and interpretation. Here, we introduce *relion_live.py* and *relion_analyse.py*, two web-based dashboards that are designed to integrate with existing *RELION* data structures. These dashboards provide a user-friendly and interactive platform for the effective visualization of *RELION* metadata. Additionally, we present a Python script integrated in *RELION* for the estimation of ice thickness and quality, enabling efficient filtration of the data set based on ice quality during movie pre-processing or in downstream processing steps. These tools empower *RELION* users to make informed decisions regarding data processing at multiple steps along the pipeline.

## 2. *relion_live.py*: a dashboard to follow data collection on the fly

Modern instruments have accelerated data acquisition considerably, easily performing the acquisition of hundreds of movies per hour thanks to aberration-free image shift and fringe-free imaging. Large fractions of the data acquired are discarded during processing due to bad quality, suboptimal ice thickness (Neselu *et al.*, 2023 ▸) or the presence of aggregated particles or partially misfolded particles (Noble *et al.*, 2018 ▸). Screening and high-end data collection time is a scarce and costly resource. Ignoring suboptimal areas or fine-tuning microscope parameters during data collection can increase the amount of useful data. However, data quality is not always evident from previsualization images. The output of the pre-processing pipeline, including beam-induced motion correction and the estimation of CTF parameters, is key to estimate this quality and can be critical to correct the course of data collection, avoiding suboptimal squares or grids.

**Figure 1:**
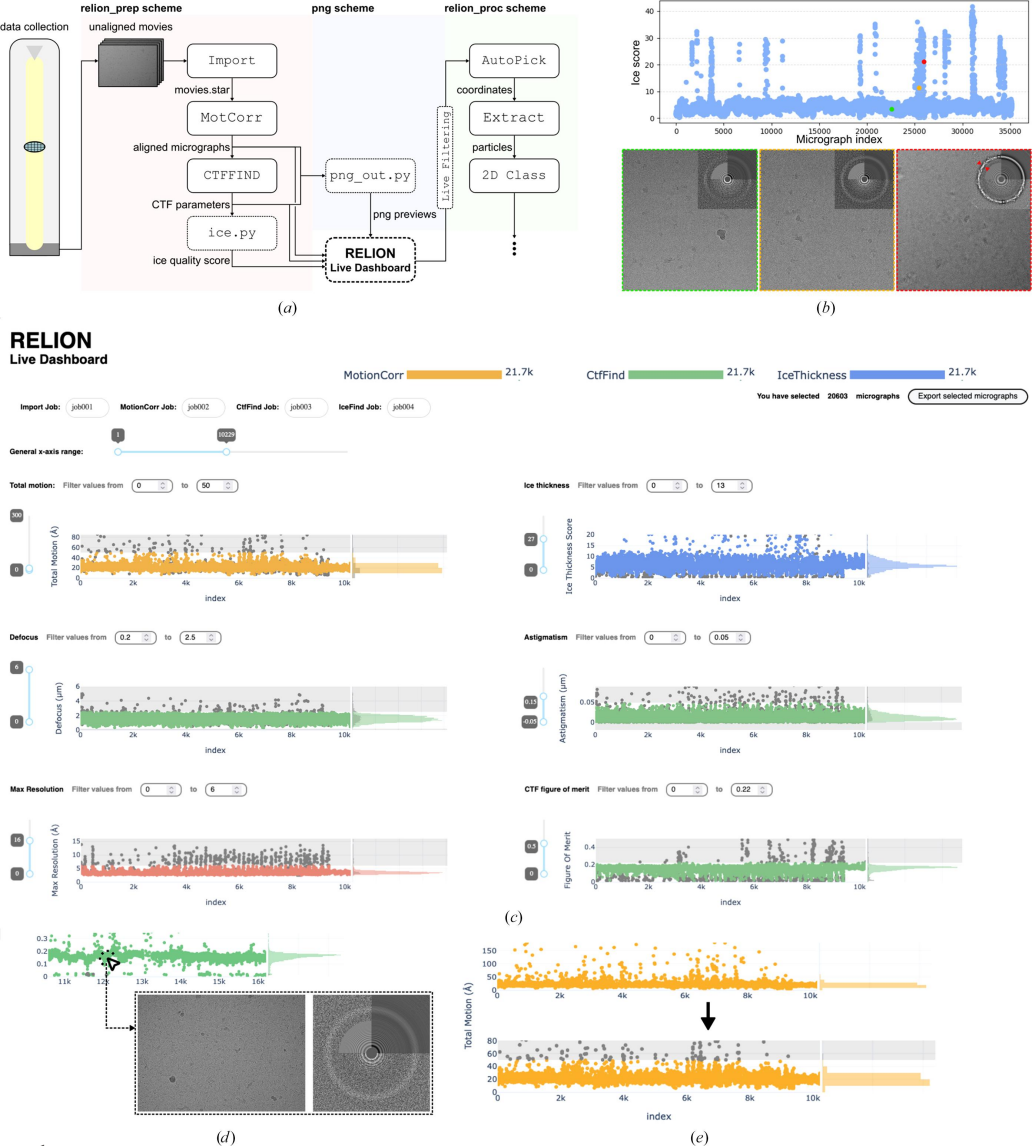
*relion_live.py*, a web-based dashboard to follow on-the-fly pre-processing. (*a*) Schematic representation of the proposed workflow for the use of *relion_live.py*. The dashed boxes indicate the steps enabled by *relion_live.py*. After starting data collection, the *relion_prep* Scheme is launched to pre-process (motion correction, CTF estimation, ice thickness estimation) batches of unaligned movies coming from the direct electron detector. Launching the *relion_png* Scheme starts *png_out.py*, which collects the averaged micrographs and CTF results to display a PNG preview of both. All results are then aggregated in the *relion_live.py* dashboard, in which thresholds are manually selected for images to be filtered on the fly. Images that are compatible with all manual thresholds are used as input for the *relion_proc* Scheme for further processing. (*b*) Ice scores of all micrographs in a data set containing 35 000 movies. The score correlates directly with the signal intensity in the spatial frequency range 1/4–1/3.6 Å^−1^ (see the arrows in the rightmost CTF inset). High-scoring micrograph clusters indicate squares with suboptimal ice thickness in the grid. (*c*) Screenshot of the appearance of the *RELION* Live Dashboard. The header contains the number of images pending processing in the selected *MotionCorr*, *CTFFIND* and *ice.py* jobs. Below, the key pre-processing results are aggregated in interactive plots. (*d*) Clicking on a point in any of the plots displays the corresponding micrograph and its estimated CTF. (*e*) It is possible to manually select thresholds for each individual parameter. In the example depicted, all micrographs with an accumulated motion, as calculated by *MotionCorr*, of over 50 Å are discarded during Live Filtering.

Several solutions have been implemented to receive live feedback on data quality in real time (Tegunov & Cramer, 2019 ▸; de la Rosa-Trevín *et al.*, 2016 ▸; Punjani *et al.*, 2017 ▸). *RELION*, however, does not have a built-in way to display this information generated during on-the-fly data processing. Driven by this need and inspired by tools such as *WARP* (Tegunov & Cramer, 2019 ▸) and *cryoSparc Live* (Punjani *et al.*, 2017 ▸), we developed *relion_live.py* (Fig. 1 ▸), a web-based interface that tracks the pre-processing output of *RELION* in real time. This tool is designed to be dependent only on the open-source software *RELION* and its native implementation for cyclic, batch processing of movies known as ‘Schemes’ (Kimanius *et al.*, 2021 ▸). *relion_live.py* aggregates the results of the beam-induced motion correction and CTF estimation steps, which provide live feedback related to the physical stability of the sample (total motion) and the optical quality of the images (estimated astigmatism, defocus and fitting of calculated CTFs) acquired by the microscope.

To complement the information provided by these calculations, we have included two tools executed within *RELION* as ‘External’ jobs: (i) a straightforward estimation of the ice thickness and quality for each individual image, named *ice.py*, and (ii) a tool to output the motion-corrected micrographs and the experimental and calculated CTFs as PNG files for their display in the dashboard, named *png_out.py*.

After setting up and starting data collection, three different Schemes are executed (Fig. 1 ▸ *a*): *relion_prep*, *relion_png* and *relion_proc*. During *relion_prep* (red in Fig. 1 ▸ *a*), *RELION* imports a batch of *N* movies directly from the direct electron detector server and feeds them into *MotionCorr* (Zheng *et al.*, 2017 ▸; Zivanov *et al.*, 2018 ▸) to perform frame alignment and dose weighting (the value of *N* will depend on the computational resources available, but *N* = ∼5–10 should provide feedback with <2 min of delay after movie acquisition using a single 48-CPU core workstation). The possibility of using the *RELION* implementation of *MotionCorr* makes the process suitable to be run in the absence of GPUs, making live pre-processing cheaper or freeing up GPU resources for further parallel downstream processing jobs. *CTFFIND*4 (Rohou & Grigorieff, 2015 ▸) uses the aligned micrographs as input and outputs an estimation of the defoci, astigmatism, CTF and different confidence scores of the micrographs for its fitting (CtfMaxResolution and CtfFigureOfMerit). These values are useful for filtering out low-quality micrographs during data acquisition, but are often insufficient. To complement this information, we included *ice.py* in the pipeline. *ice.py* is a computationally inexpensive method to estimate ice quality for a given micrograph as well as the presence of crystalline ice. One of the default *CTFFIND*4 outputs is the radially averaged signal of the micrograph power spectrum. We take advantage of this calculation and use the average of the signal in the spatial frequency range 1/4–1/3.6 Å^−1^, in which both vitreous and crystalline ice show intensity maxima (Dubochet & McDowall, 1981 ▸; McMullan *et al.*, 2015 ▸) as a proxy for ice thickness and crystalline ice contamination. While the resulting score integrates multiple sources for the increase in ice signal and does not correlate directly with any physical magnitude, it is useful to identify low-quality ice without the need for manual inspection. When plotted against the collection time, high-scoring micrographs cluster together, suggesting regions of the grid containing thick ice (Fig. 1 ▸ *b*). This score is included as metadata labelled MicrographIceThickness, allowing further analysis or the selection of particles based on specific ice thickness during subsequent steps of data processing. The *relion_png* Scheme runs in parallel, piping aligned movies from *MotionCorr* and the calculated CTF images into *png_out.py* to produce previews of both as PNG images that are easily displayed by the web-based dashboard (blue in Fig. 1 ▸ *a*).

Key parameters for judging micrograph quality derived from the *MotionCorr* (total motion), *CTFFIND* (astigmatism, defocus, max resolution and figure of merit) and *ice.py* (rice thickness) jobs are aggregated and displayed in the *relion_live.py* web-based dashboard (Fig. 1 ▸ *c*). Its plots populate as cycles of *relion_prep* end, allowing immediate data-quality feedback. Each individual point in the scatter plots is linked to its own *png_out.py* output. Clicking on the scattered points will display a preview of the individual micrograph and the corresponding CTF, allowing easy and immediate correlation between estimated optical parameters and the appearance of the micrograph (Fig. 1 ▸ *d*).

The scatter plots aggregated in *relion_live.py*, presenting optical parameters as a function of time of movie acquisition, allow the visual identification of micrographs that are clear outliers, as well as clusters of outliers representing bad areas of the grid during automated data collections (Fig. 1 ▸ *b*). The user can also filter out low-quality images during data collection by manually adding custom thresholds to each parameter (Fig. 1 ▸ *e*). The micrographs that are filtered out will not be imported or processed further, removing useless data from the on-the-fly processing by the *relion_proc* Scheme (green in Fig. 1 ▸ *a*; Supplementary Video S1).

The fact that *relion_live.py* is a web-based app means that it can also be provided to users to follow data processing in a service environment. It is also important to note that even though *relion_live.py* is designed to be used to follow data pre-processing on the fly, it can be used to filter previously pre-processed data sets in an efficient manner.

## 3. *relion_analyse.py*: integrating and visualizing *RELION* metadata

Single-particle cryo-EM processing has been streamlined to allow structure determination of well behaved samples even to high resolution in a nonsupervised manner (Cushing *et al.*, 2023 ▸). However, most projects still rely on intensive and time-consuming expert manual labour to yield interpretable results due to the intrinsic difficulties of the sample or to reach the full potential of a data set in terms of attaining high resolution or accounting for the full extent of its heterogeneity. In addition, the parameters used internally by the software are not readily accessible from their respective GUIs, and their interpretation and manipulation only remain possible via command-line interventions. Both aspects of data processing drive a wedge between newcomers or occasional cryo-EM users and the full potential of the technique. The appearance of graphical interfaces dedicated to different software packages (de la Rosa-Trevín *et al.*, 2016 ▸; Punjani *et al.*, 2017 ▸; Fernandez-Leiro & Scheres, 2017 ▸; Grant *et al.*, 2018 ▸; Moriya *et al.*, 2017 ▸), including *Doppio*, a new project by *CCP-EM* to provide a global graphical environment for *RELION* and the rest of the *CCP-EM* suite, is paving the way for this user profile to employ single-particle cryo-EM as an additional tool in their research. Trying to facilitate access to the metadata and its analysis, we have developed *relion_analyse.py*, a web-based dashboard for plotting and interacting with the data stored in *RELION* STAR files. This dashboard is a collection of graphical tools that allow the plotting of any metadata present in the underlying STAR files to easily identify trends in the data and to troubleshoot difficult data sets.

**Figure 2:**
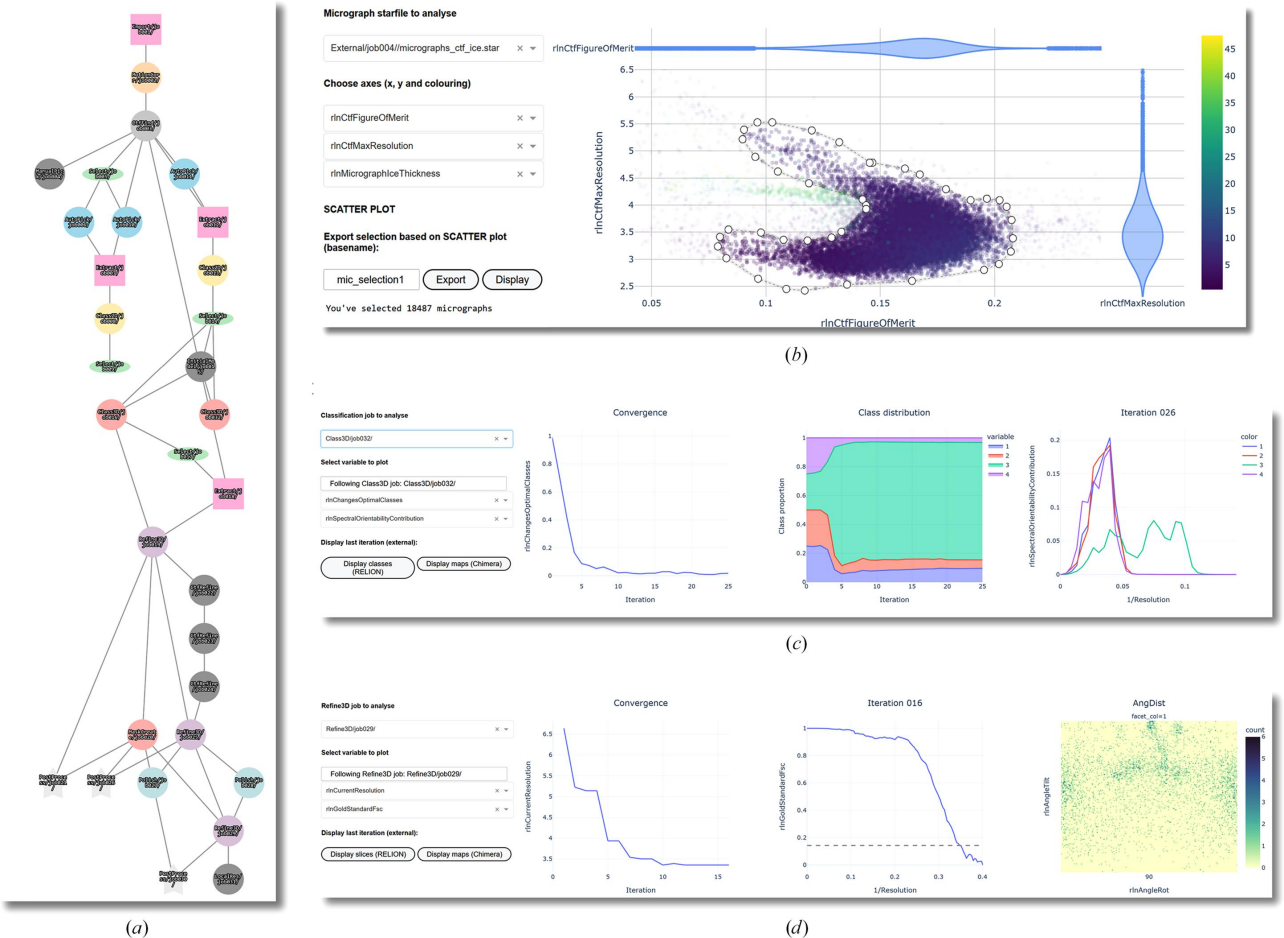
*relion_analyse.py*, a web-based dashboard for *RELION* metadata analysis. (*a*) The ‘RELION Pipeline’ tab depicts a *RELION* project as an interactive graph in which nodes represent jobs and vertices represent input/output relationships between them. (*b*) The ‘Analyse Micrographs’ and ‘Analyse Particles’ tabs contain a plot where three variables in any STAR file in the *RELION* project can be represented simultaneously. The plot is interactive, allowing zooming and panning. The lasso tool implemented in Dash graphs allows the manual selection of images to be exported as an independent STAR file. They can then be readily imported back into *RELION* for further processing. (*c*) The ‘Follow 2D Classification’ and ‘Follow 3D Classification’ tabs allow 2D and 3D classification jobs to be followed as they run. They present a ‘Convergence’ plot (left plot), in which parameters such as ChangesOptimalClasses or OverallAccuracyRotations, which typically decrease over iterations if the job is successful, can be plotted. The ‘Class Distribution’ plot (middle) represents the proportion of particles in each class across iterations. In the example shown above it is noticeable that the classification of particles does not change significantly after iteration 10. The last plot (right), which is only present for the 3D case, represents SpectralOrientabilityContribution per class, which provides an estimate of which spatial frequencies contribute more to the alignment. (*d*) The ‘Follow 3D Refine’ tab contains a ‘Convergence’ plot, a representation of the FSC curve from the most recent refinement iteration, and a heatmap of the angular distribution of the particles.

The first of the tools aggregated in *relion_analyse.py* is ‘RELION Pipeline’, an interactive graphical summary of the current *RELION* project. It depicts a *RELION* project as an interactive graph in which nodes represent each individual job and vertices represent input/output relationships between two nodes. It allows the processing jobs to be visualized in a tree-like manner to understand which inputs and outputs are related to each job. It also displays the collection of job parameters used for any given node in the graph, allowing a quick analysis of the jobs and processing strategy (Fig. 2 ▸ *a*).

The two following tabs, ‘Analyse micrographs’ and ‘Analyse particles’, plot each individual image, either motion-corrected micrographs or individual particles, as scattered points using their corresponding metadata. After selecting a given job for display, up to three variables can be chosen, corresponding to any of the metadata labels in the corresponding STAR files, to plot the individual images. This representation of data sets allows the identification of problematic parts of the data set, as experimental images tend to cluster in groups with similar imaging conditions. The interactive capabilities of these plots allow custom in-plot filtering of data using the lasso tool for the further processing or evaluation of only a subset of selected images rather than using sequential one-dimensional thresholds (Fig. 2 ▸ *b*). The selected images are exported as a STAR file that can be readily imported back into *RELION* for further processing. In the case of the ‘Analyse micrographs’ tab, clicking any of the points in the graph displays the corresponding micrograph and CTF, enabling a quick understanding of the data set and the characteristics of the images depending on different optical parameters.

The three following tools, ‘Follow 2D Classification’, ‘Follow 3D Classification’ and ‘Follow 3D Refinement’, are meant to be used as dashboards where the fundamental information of the output of a job is collected and visually summarized. The information is displayed in the dashboard after each refinement cycle finishes, not only upon job completion, so that jobs can be followed live. All of the tools include a ‘Convergence’ plot, in which either the variables ChangesOptimalClasses for 2D and 3D classifications or CurrentResolution for 3D refinements are plotted by default as a function of the cycle number (Fig. 2 ▸ *c*). We find this approach useful to stop running jobs that are rendering useless results due to bad parametrization of the run or bad data quality, or jobs that achieve convergence before the last iteration programmed, thus optimizing the use of computational resources and limiting energy consumption. Any other metadata value in the optimizer.star files can be plotted against iteration number, facilitating the analysis of trends within the run. Particle distribution changes through iterations are also plotted for the 2D and 3D classification jobs (Fig. 2 ▸ *c*), facilitating analysis of the classification performance and convergence, and troubleshooting classification issues. For 3D classification and 3D refinement jobs, we also include a plot with the data from the model.star files from the latest iteration available, allowing the graphical inspection of parameters such as GoldStandardFsc, SpectralOrientabilityContribution, FourierCompleteness or SsnrMap (Figs. 2 ▸ *c* and 2 ▸ *d*). 3D classification and 3D refinement jobs also display a 2D heatmap of particle angular assignments (rotation versus tilt) to evaluate particle orientations (Fig. 2 ▸ *d*). For the inspection of 2D classes and volumes, *relion_display* and *Chimera* (Goddard *et al.*, 2018 ▸) can be executed directly from these tabs (Supplementary Video S2).

**Figure 3:**
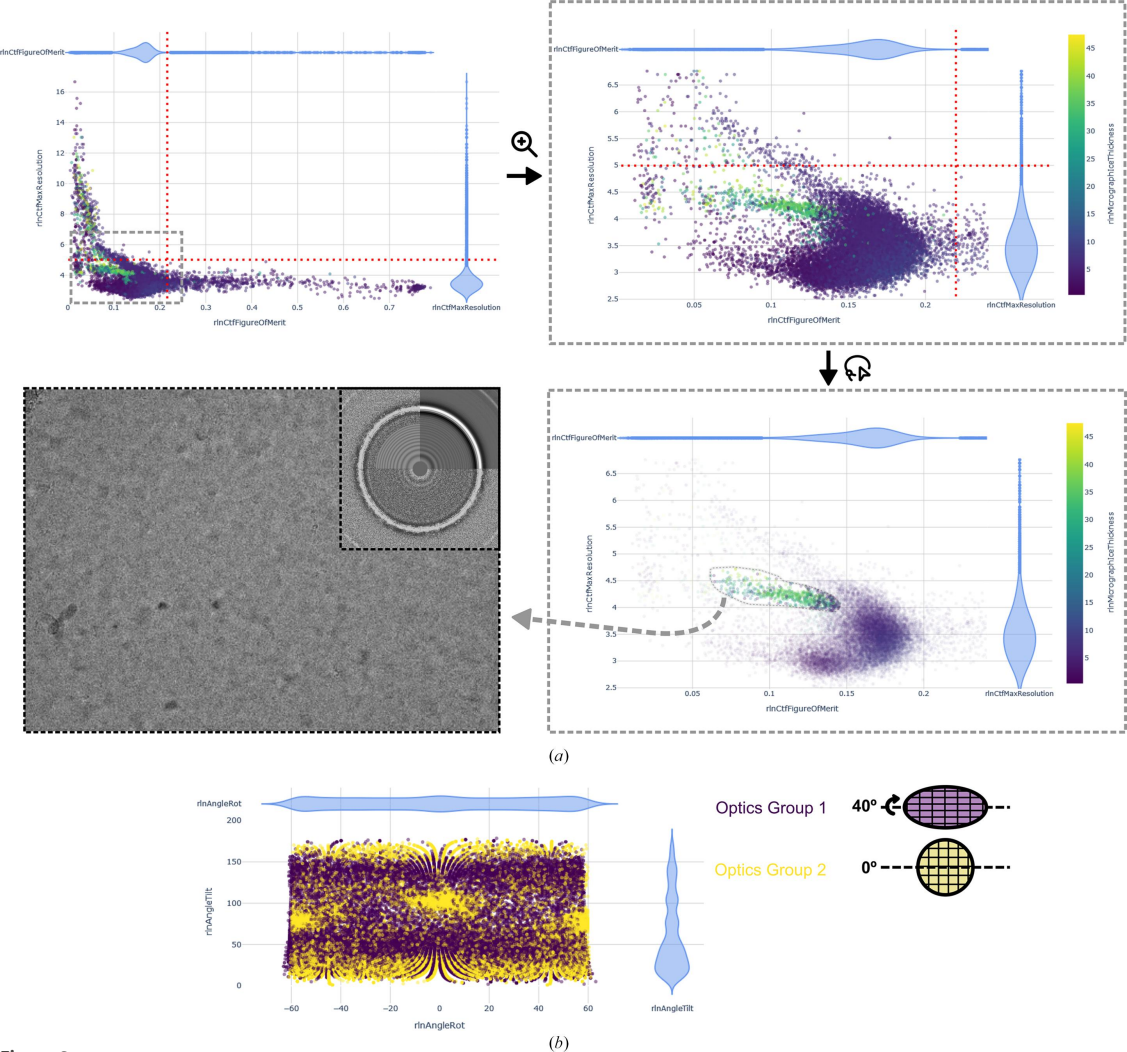
Representative use cases of the ‘Analyse Micrographs’ and ‘Analyse Particles’ tabs in *relion_analyse.py*. (*a*) After pre-processing (motion correction and CTF estimation) of a data set, a common approach is to manually select thresholds to discard low-quality micrographs. The red dotted lines represent sensible thresholds for CtfMaxResolution and CtfFigureOfMerit. Colouring the plot by ice score (calculated by *ice.py*) shows a group of micrographs with low-quality ice. These would be included for further processing with the manual thresholds but can be easily removed using the lasso tool. (*b*) Angular distribution of particles (AngleTilt versus AngleRot) resulting from merging data sets collected at 0° and 40° tilt, respectively, and plotted in the ‘Analyse Particles’ tab. Colouring by OpticsGroup, we can observe that the tilted data set (optics group 1, purple) fills up the orientational space, providing views that are absent in the 0° data set (optics group 2, yellow). Data were obtained from EMPIAR-10096 and EMPIAR-10097 (Tan *et al.*, 2017 ▸).

One common scenario illustrating the usefulness of this tool is the first few steps of a processing pipeline. The most usual approach is to set an upper threshold of the CtfMaxResolution parameter estimated by *CTFFIND* and let the classification and refinement algorithms take care of discarding low-quality images. However, filtering the set of micrographs more extensively can easily be performed when several parameters are represented together in the Analyse Micrographs tab. In Fig. 3 ▸(*a*), the red dotted lines represent sensible thresholds that could be used to filter micrographs during pre-processing. Plotting CtfMaxResolution versus CtfFigureOfMerit, several clusters of micrographs are evident, and all of them would fall within the limits of the manually selected thresholds. Adding colour as a function of a third variable, MicrographIceThickness, calculated by *ice.py* for each micrograph, we realize that one particular group of micrographs represent low-quality areas with non­vitreous ice that can be immediately discarded from further processing, avoiding the inclusion of suboptimal particles from the earliest stages of the project (Fig. 3 ▸ *a*). Fig. 3 ▸(*b*) showcases another example, using the ‘Analyse Particles’ tab to display the Euler angles assigned to particles during 3D refinement by *RELION* and to represent the orientation distribution in the form of a scatter plot. Preferential orientation of particles is one of the most common limitations in cryo-EM SPA to achieving interpretable 3D reconstructions of macromolecules (Glaeser & Han, 2017 ▸). In a common approach to increase the diversity of views, the sample is tilted in the microscope stage to image the particles in different orientations and merge them with untilted data in a single set of particles (Tan *et al.*, 2017 ▸). While the orientation distribution is also provided natively by *RELION* as .bild files produced by the 3D Refinement jobs, our tool allows a third variable to be represented as colours. By using OpticsGroupNumber as the third variable, we identify particles coming from each data set and observe how the tilted data set provides new projections that efficiently fill the orientational space gap (Fig. 3 ▸ *b*). Examples of the use of the Follow 2D and 3D Classification tabs include the efficient troubleshooting of common pitfalls during classification, such as runs that collapse into a single class, runs converging in early iterations, runs that do not converge, 3D classifications that separate classes based on orientations or defoci, *etc.*

## 4. Discussion

Here, we introduce two tools, *relion_live.py* and *relion_analyse.py*, that are designed to enhance the capabilities of cryo-EM data processing. These tools are intended to streamline cryo-EM data-analysis practices and provide an organized structure for day-to-day activities.

*relion_live.py* provides a web-based dashboard enabling users to monitor data collection in real time, helping them to make informed decisions during data acquisition. It offers insights into the quality of the acquired micrographs and allows on-the-fly filtering, ultimately optimizing the utilization of valuable data-collection time. This tool can easily be incorporated into the *relion_it.py* script to start the *RELION* on-the-fly processing pipeline, providing a dashboard to follow data processing in real time from the beginning of data collection.

Secondly, *relion_analyse.py* offers a web-based platform for visualizing and analysing the cryo-EM metadata generated during data processing. Being able to easily visualize different parameters from STAR files coming from different jobs in the project allows the user to understand the behaviour of the runs and quickly identify potential sources of error to re-route the workflow. It simplifies the interpretation of complex data and facilitates troubleshooting, making it a valuable resource for both experienced researchers and non-expert users.

An unexpected advantage of the use of these tools is their value as educational resources, allowing expert and non-expert users to better understand the algorithms and parameters behind SPA data processing.

## 5. Brief notes on implementation

Both tools are written in Python and use Dash from Plotly (Hossain, 2019 ▸) for the aggregation of plots and their deployment as a web-based application. The internal STAR files are parsed and written using the *starfile* Python package (Burt, 2020 ▸) and filtered using the *pandas* package. While the dashboard responsiveness is fast for most projects, projects containing several hundreds of jobs take longer to load. However, after initial loading both *relion_live.py* and *relion_analyse.py* function fluently. Handling STAR files with millions of particles poses a challenge due to the sheer volume of data points for plotting. In such cases, an effective strategy is to consider removing the third axis option (colour). As data sets grow larger, this issue is likely to become increasingly common. Consequently, we are actively working on addressing this challenge as part of our ongoing efforts. Both tools are directly compatible with currently available versions of *RELION*4 (Kimanius *et al.*, 2021 ▸) and later versions (Schwab *et al.*, 2023 ▸; Kimanius *et al.*, 2023 ▸). Future compatibility of this tool is straightforward as it feeds from the *RELION* project structure and pipeline.star file. New job types and parameters are automatically read from these files and included. Thanks to the modularity of the code, all of the tools presented in this manuscript could easily be integrated into larger projects that effectively function as GUIs for *RELION*, such as *Doppio* or *Scipion* (de la Rosa-Trevín *et al.*, 2016 ▸).

Installation of these tools is straightforward by following the steps at https://github.com/cryoEM-CNIO/CNIO_Relion_Tools. Once installed, the dashboards can be executed by typing relion_live.py from within the root folder of a *RELION* directory. By default, ports 8050 and 8051 are used for *relion_live.py* and *relion_analyse.py*, respectively, but they can conveniently be changed by adding the argument --port during the launch of the app. *ice.py* and *png_out.py* are called directly from the *RELION* GUI as an external job. We provide our Schemes folder and *relion_it.py* configuration file as an example for the implementation of these tools within the pre-processing *RELION* pipeline.

## 6. Data availability

All of the tools described in this manuscript can be found at https://github.com/cryoEM-CNIO/CNIO_Relion_Tools.

## Supplementary Material

Supplementary Video S1. relion_live.py, a web-based dashboard to follow on-the-fly pre-processing. The relion_live.py dashboard aggregates the pre-processing results of the relion_prep scheme. By clicking on each of the scattered points, the micrograph and its corresponding CTF estimation are displayed. The video illustrates how, by interacting with the plots, a data set can be explored using the display gallery. Applying manual thresholds allows micrographs that mostly contain carbon foil or bad-quality ice to be discarded. DOI: 10.1107/S2059798323010902/ic5122sup1.mp4

Supplmentary Video S2. Volumes and classes display via relion_display or Chimera from the relion_analyse.py dashboard. When inspecting a 2D or 3D Classification or 3D Refinement job, the classes and volumes can be readily displayed using relion_display or the local installation of Chimera or ChimeraX. DOI: 10.1107/S2059798323010902/ic5122sup2.mp4
